# Putative Membrane-Bound Transporters MFSD14A and MFSD14B Are Neuronal and Affected by Nutrient Availability

**DOI:** 10.3389/fnmol.2017.00011

**Published:** 2017-01-25

**Authors:** Emilia Lekholm, Emelie Perland, Mikaela M. Eriksson, Sofie V. Hellsten, Frida A. Lindberg, Jinar Rostami, Robert Fredriksson

**Affiliations:** ^1^Department of Neuroscience, Functional Pharmacology, Uppsala UniversityUppsala, Sweden; ^2^Department of Pharmaceutical Biosciences, Molecular Neuropharmacology, Uppsala UniversityUppsala, Sweden

**Keywords:** MFSD14A, HIAT1, MFSD14B, HIATL1, SLC, MFSD, transporter protein

## Abstract

Characterization of orphan transporters is of importance due to their involvement in cellular homeostasis but also in pharmacokinetics and pharmacodynamics. The tissue and cellular localization, as well as function, is still unknown for many of the solute carriers belonging to the major facilitator superfamily (MFS) Pfam clan. Here, we have characterized two putative novel transporters MFSD14A (HIAT1) and MFSD14B (HIATL1) in the mouse central nervous system and found protein staining throughout the adult mouse brain. Both transporters localized to neurons and MFSD14A co-localized with the Golgi marker Giantin in primary embryonic cortex cultures, while MFSD14B staining co-localized with an endoplasmic retention marker, KDEL. Based on phylogenetic clustering analyses, we predict both to have organic substrate profiles, and possible involvement in energy homeostasis. Therefore, we monitored gene regulation changes in mouse embryonic primary cultures after amino acid starvations and found both transporters to be upregulated after 3 h of starvation. Interestingly, in mice subjected to 24 h of food starvation, both transporters were downregulated in the hypothalamus, while *Mfsd14a* was also downregulated in the brainstem. In addition, in mice fed a high fat diet (HFD), upregulation of both transporters was seen in the striatum. Both MFSD14A and MFSD14B were intracellular neuronal membrane-bound proteins, expressed in the Golgi and Endoplasmic reticulum, affected by both starvation and HFD to varying degree in the mouse brain.

## Introduction

Membrane-bound transporter proteins play an important role in cell survival, as these proteins allow interaction between the external and internal environment of cells, importing and exporting substances across the lipid barrier. The transport of substances across membranes has important implications, not just for homeostasis, but also for pharmacokinetic and the safety and efficacy of drugs (Rask-Andersen et al., 2013; Lin et al., 2015). Transporters are a class of proteins that are underrepresented in research (Cesar-Razquin et al., 2015) and many of the identified transporters remain orphans in that their localization and/or function remains unknown. Classifying the vast number of transporters is traditionally done by grouping them into families based on structure and/or function (Hediger et al., 2004, 2013; Fredriksson et al., 2008; Schlessinger et al., 2013), and the two largest superfamilies of transporters are the ATP-binding cassette (ABC) superfamily and the solute carrier (SLC) superfamily. The SLCs are facilitated or secondary active transporters (Fredriksson et al., 2003), hence working independently of ATP. In humans, they are a diverse group of proteins, consisting of 456 members divided into 52 subfamilies (Hediger et al., 2013; Cesar-Razquin et al., 2015), located in the outer plasma membrane or internal organelles membranes. Many are conserved in prokaryotes, invertebrates, and vertebrates indicating once again their importance, or at the very least, their important past.

Based on sequence similarities, two orphan putative transporters MFSD14A and MFSD14B have previously been classified as atypical SLCs belonging to the major facilitator superfamily (MFS) Pfam clan (Sreedharan et al., 2011). The proteins were first named according to large cDNA sequencing projects as hippocampus abundant transcript 1, HIAT1, and hippocampus abundant transcript like 1, HIATL1 (Fredriksson et al., 2008) but was later on renamed to MFSD14A and MFSD14B. The MFS is a diverse family, and despite shared similar functions, they share weak sequence similarities between members in conventional linear sequence alignment studies (Madej et al., 2013). MFS proteins are present in species from bacteria to eukaryotes (Lemieux, 2007), and both MFSD14A and MFSD14B are conserved in several, including mice and *Drosophila melanogaster* (Pao et al., 1998; Sreedharan et al., 2011). *Mfsd14a* encodes a protein of 490 amino acids, revealing similarities to sugar transporters due to the sugar transporter specific motif found between the second and third putative transmembrane domains (Gerhard et al., 2004). Northern hybridization experiments performed on mouse brain tissue reveal an even mRNA distribution of *Mfsd14a* from embryonic day 13 into adulthood (Matsuo et al., 1997). *Mfsd14b* encodes a protein slightly larger than MFSD14A, at 506 amino acids (Humphray et al., 2004). Similar to MFSD14A, MFSD14B is thought to have 12 transmembrane regions (Ota et al., 2004) which is the common amount of transmembrane regions for most MFS members (Law et al., 2008). In addition to expression in hippocampus, both *Mfsd14a* and *Mfsd14b* reveals gene expressions in peripheral rat tissues, with high *Mfsd14a* levels in the testis and high *Mfsd14b* expression in the skeletal muscles (Sreedharan et al., 2011). The function of neither MFSD14A nor MFSD14B is known and neither is the cellular protein localization. However, MFSD14A was recently implicated to be involved in spermatogenesis as mutations in the *Mfsd14a* gene causes infertile mice with dysmorphic sperm head formation and abnormal nuclear condensation (Doran et al., 2016). Here we performed histological characterization of both MFSD14A and MFSD14B, focusing on protein localization in the central nervous system in mice. Furthermore, we investigated involvement in energy homeostasis by screening for gene expression changes in primary cultures exposed to amino acid starvation, and in mice subjected to different diets. Both transporters were found to change expression levels due to starvation in both primary cultures and *in vivo* samples as well as due to high fat diet (HFD). Using immunohistochemistry, we found both MFSD14A and MFSD14B staining throughout the adult mouse brain. Both transporters were found to be expressed in neurons with intracellular staining patterns co-localized to the Golgi complex and endoplasmic reticulum (ER).

## Results

### Both MFSD14A and MFSD14B Are Atypical SLCs

**FIGURE 1:**
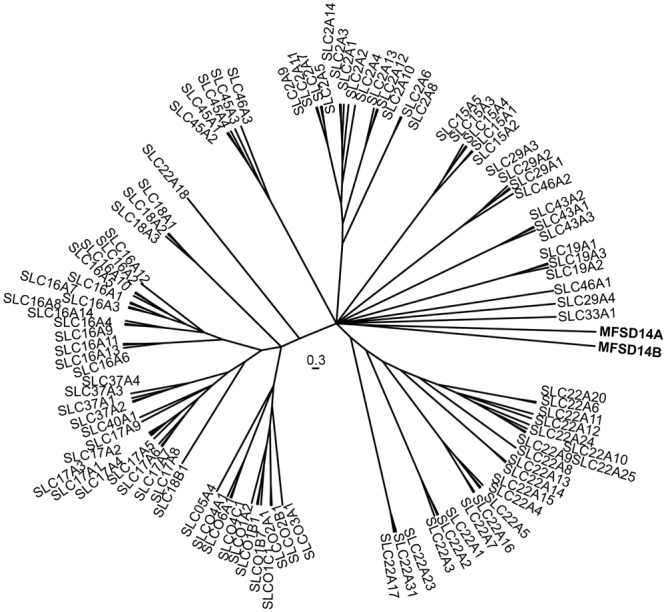
**Phylogenetic analysis of solute carriers (SLCs).** The interrelation between the human MFSD14A and MSFD14 and “SLCs of MFS type” proteins sequences were calculated using a Bayesian approach and depicted as a phylogenetic branching tree. MFSD14A and MFSD14B do not cluster to any of the other families, but have their own branches from the central node.

We combined the sequences of MFSD14A and MFSD14B with SLCs of MFS clan to study their phylogenetic relationships. The outcome (**Figure 1**) placed MFSD14A and MFSD14B with a shared closest branching node with several SLC families (15, 19, 22, 29, and 43). However, both MFSD14A and MFSD14B had their own branch in the tree projecting out from their common node, meaning that they differed to such extent that they cannot be grouped into any existing MFS family when classified according to the SLC tables^1^. The sequence similarity between human MFSD14A and MFSD14B was high, at 75.4%, as revealed by pairwise global protein alignment using EMBOSS needle and even higher, at 85.6%, when focusing on protein domains. Furthermore, both proteins were seen to be very similar between mouse and human, with a 100% sequence similarity for MFSD14A and 93.8% similarity for MFSD14B^2^ (Rice et al., 2000).

### Gene Expression of *Mfsd14a* and *Mfsd14b* throughout Most of the Brain and Peripheral Tissues of Adult Mice

**FIGURE 2:**
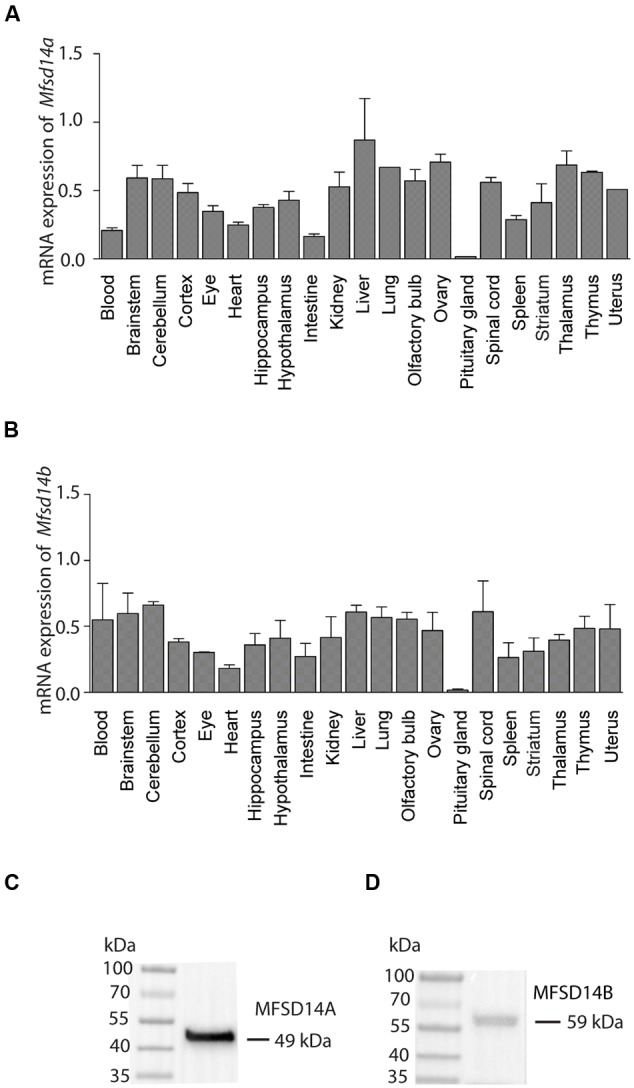
**mRNA expression of *Mfsd14a* and *Mfsd14b* in mouse tissue.** qRT-PCR analysis of mRNA expression in brain and peripheral tissue samples from adult C57Bl6/J mice. The mRNA expression was normalized using four stable housekeeping genes (*bTub, Rpl19, Cyclo*, and *Actb)*, the tissue with the highest gene expression for each transporter was set to 100% and the relative expression levels of the tissue plotted (±SD). **(A)** Relative expression of *Mfsd14a* with the highest levels in liver set to 100%. **(B)** Relative expression of *Mfsd14b* with the highest levels in liver set to 100%. Verification of antibodies used in the histological methods was performed using Western Blot with a protein preparation made from mouse brain. **(C)** Staining for MFSD14A gives a band at 49 kDa (expected size at 53 kDa) and **(D)** staining for MFSD14B gives a band at 59 kDa (expected size at 55 kDa).

To characterize gene expression levels of both transporters, a tissue panel of a selection of brain and peripheral tissue from wild type mice was used. Both *Mfsd14a* and *Mfsd14b* were expressed in most tissues and areas of the brain (**Figure 2**) except for the pituitary gland where a much lower relative expression was found for both transporters. The liver gave the highest relative expression for both transporters. For *Mfsd14a*, a relatively even expression levels were seen (**Figure 2A**) while for *Mfsd14b*, the gene expression was more varied in the tissues included in the screen (**Figure 2B**).

### Verification of Antibodies for Protein Localization Studies

Commercially available antibodies for both MFSD14A and MFSD14B were used for protein localization and were verified using Western Blot on protein samples prepared from mouse brain tissue. Following chemiluminescent detection, one band for MFSD14A at 49 kDa, with the expected size at 53 kDa (MGI:1201609) (Cunningham et al., 2015) (Ensembl release 82) could be found (**Figure 2C**). For MFSD14B, one band at 59 kDa was found which correlated to the expected 55 kDa size (MGI:1913881) (Cunningham et al., 2015) (Ensembl release 82) (**Figure 2D**). Membrane proteins are known to bind more SDS and therefore migrate faster on the gel than globular proteins (Rath et al., 2009), and the slightly lower kDa found for MFSD14A was hence expected. MFSD14B gave a somewhat larger band that expected, which could be due to the 13 O-GalNAc glycosylation sites predicted to be present on the protein, as found by analysis via NetOGlyc 4.0 (Steentoft et al., 2013). The same prediction analysis returned only five such sites for MFSD14A.

### MFSD14A and MFSD14B Are Found in Intracellular Organelles in Neurons throughout the Adult Mouse Brain

**FIGURE 3:**
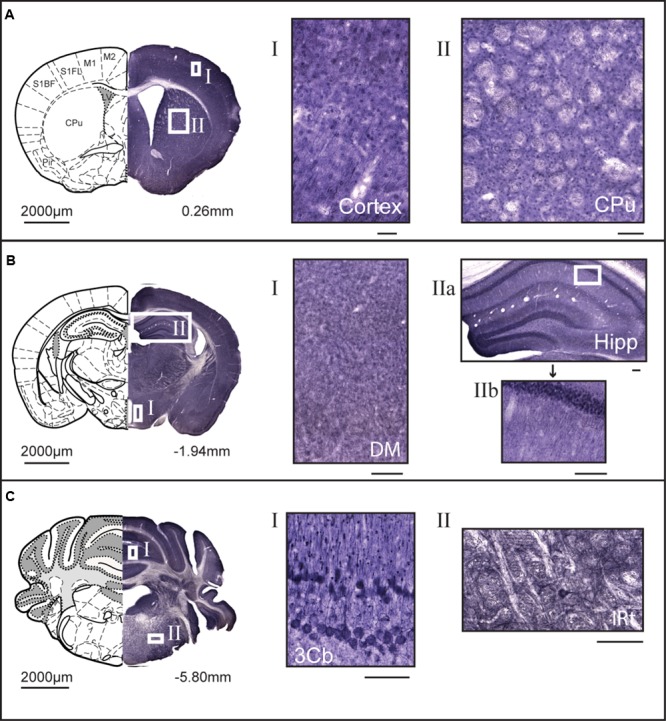
**3,3′-diaminobenzidine (DAB) immunohistochemistry of MFSD14A in the mouse brain.** Seventy micrometers of coronal sections from adult mice brains were stained for MFSD14A. Schematic brain atlas description was modified from the 2015 Allen Institute for Brain Science. Allen Mouse Brain Atlas available from http://mouse.brain-map.org (Lein et al., 2007). **(A)** Brain section from bregma 0.26 mm with close ups from areas of the cortex and striatum show abundant staining of cell bodies. **(B)** Brain section from bregma -1.94 mm with close ups from hippocampus, and from around the third ventricle, including areas of the hypothalamus. Abundant staining was found in all areas of the cortex, the piriform hippocampus, including dentate gyrus, and areas around both the dorsal third ventricle and the third ventricle. **(C)** Brain section from bregma -5.80 mm, with close ups of heavily stained cells in the third cerebellar lobule and large cells in intermedial reticular nucleus. The scale bars for the adjacent magnifications represent 100 μm.

**FIGURE 4:**
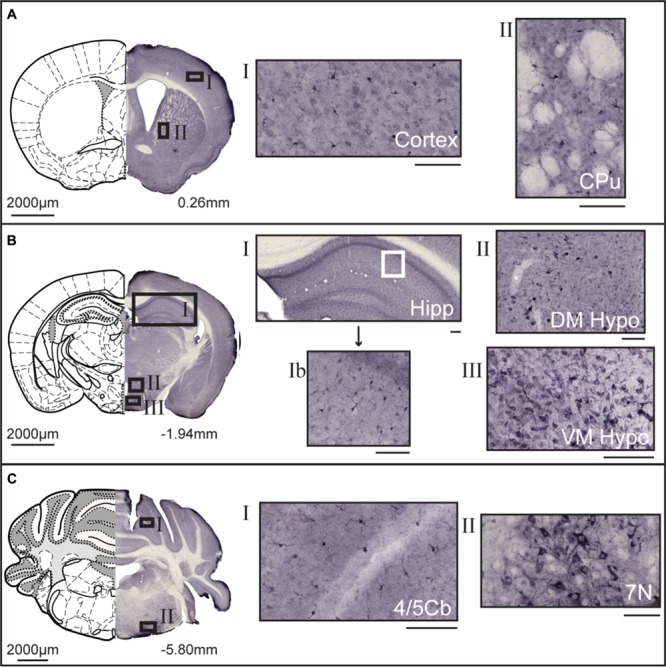
**DAB immunohistochemistry of MFSD14B in the mouse brain.** Seventy micrometers coronal sections from adult mice brains were stained with anti-HIATL1 antibody (SAB2107506, Sigma-Aldrich). Schematic brain atlas description was modified from the 2015 Allen Institute for Brain Science. Allen Mouse Brain Atlas available from http://mouse.brain-map.org (Lein et al., 2007). **(A)** Brain section from bregma 0.26 mm with close ups from areas of the cortex and striatum show staining of scattered cells. Both cell bodies as well as projections are stained. **(B)** Brain section from bregma -1.94 mm with close ups from hippocampus, and from around the third ventricle, including areas of the hypothalamus. Stained cells were found scattered in all areas of the cortex, hippocampus, and thalamus. Around both the third ventricle larger cells with clear projections were found. **(C)** Brain section from bregma -5.80 mm, with close ups of cerebellar lobule and large heavily stained cells in the facial nucleus. The scale bars for the adjacent magnifications represent 100 μm.

Non-fluorescent free floating immunohistochemistry on 70 μm thick brain sections were performed for MFSD14A and MFSD14B to provide an overview of the protein expression pattern throughout the adult mouse brain. Staining could be seen throughout the brain, and not just in the hippocampus, despite their given names. MFSD14A was found in abundance (**Figure 3**) and representative close ups from cortex and caudate putamen (striatum) can be seen in **Figure 3A**. Slight differences in staining patterns could be seen for cells in different parts of the brain, for instance, a punctuated staining pattern was found in the anterior sections while more projections were seen in areas in more posterior parts. At bregma -1.94, a high density of stained cells was found in the cortex, piriform cortex, hippocampus and dentate gyrus (**Figure 3B**). Around the third ventricle a lower density of cells were stained (**Figure 3B**). Moving rostral, to bregma -5.80, MFSD14A staining was prominent in cerebral lobules (**Figure 3C**). Large cells stained for MFSD14A could be found in the reticular nucleus, as shown in close up in **Figure 3C** II. Staining for MFSD14B could also be found throughout the brain, however, at a lower density (**Figure 4**). In addition, staining including both cell body and projections can be seen in the close ups (**Figures 4A–C**). Compared to the staining for MFSD14A, MFSD14B displayed a much more scattered staining pattern with a much lower density of cells stained. Staining for MFSD14B was found in areas throughout the cortex, striatum (**Figure 4A**), around the hippocampus, around the third ventricle and hypothalamus (**Figure 4B**). Staining was also found in the lobules of the cerebellum (**Figure 4C**), and prominent staining of large cells were found in the facial nucleus, as seen in the close up in **Figure 4C**.

**FIGURE 5:**
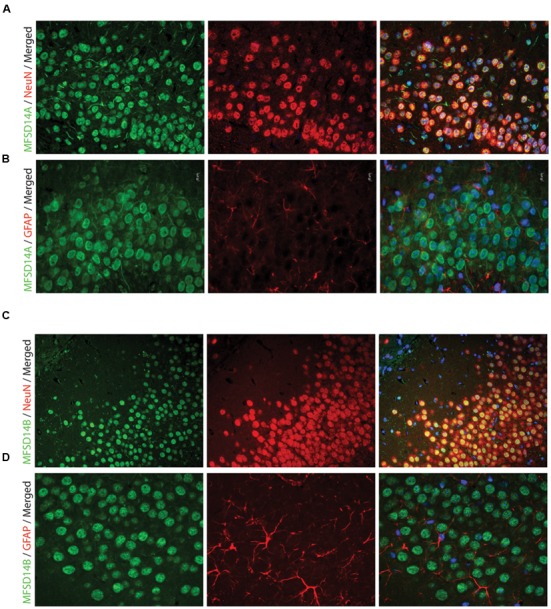
**Fluorescent immunohistochemistry of MFSD14A and MFSD14B in the mouse brain.** Seven micrometers paraffin embedded adult mouse brain sections were stained for MFSD14A or MFSD14B. Representative staining patterns were captured from areas of the hippocampus. **(A)** MFSD14A staining (green) combined with DAPI nuclear marker (blue) neuronal marker NeuN (red) or **(B)** astrocytic marker GFAP (red). Co-localization was seen with neurons and not with astrocytes. **(C)** MFSD14B staining (green), DAPI nuclear marker (blue) combined with neuronal marker NeuN (red) or **(D)** astrocytic marker GFAP (red). Co-localization was seen with neurons and not with astrocytes.

**FIGURE 6:**
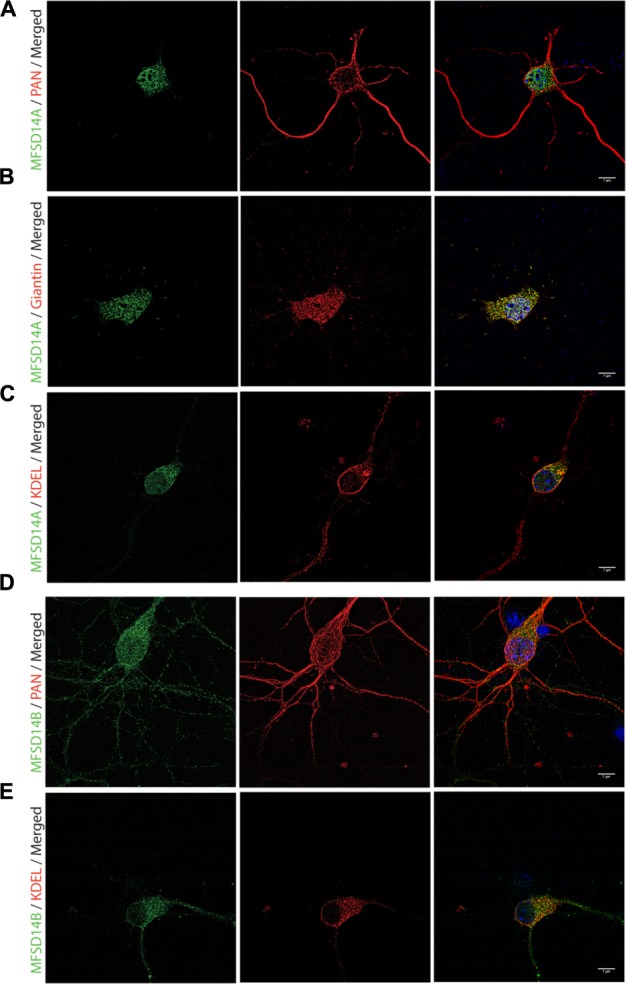
**Fluorescent immunocytochemistry of MFSD14A and MFSD14B in primary cortex cultures.** Mouse cortex primary cultures from e14.5-15 were stained for MFSD14A and MFSD14B together with intracellular markers. MFSD14A staining (green) combined with **(A)** Pan neuronal staining (red), **(B)** anti-Giantin (red) staining for Golgi localization and **(C)** anti-KDEL staining for ER localization. MFSD14B staining (green) combined with **(D)** Pan neuronal staining (red) and **(E)** anti-KDEL staining (red). DAPI nuclear marker (blue) in all merged pictures.

To further study, the distribution of these proteins in specific cell types, immunohistochemistry on mouse paraffin sections was performed. Throughout the brain, both MFSD14A and MFSD14B co-localized with the common neuronal nuclei marker NeuN (Mullen et al., 1992; Sarnat et al., 1998), but not with the astrocyte marker GFAP (Reeves et al., 1989) (**Figures 5A–D**). Here, as in the non-fluorescent immunohistochemistry, the staining patterns for both transporters were ubiquitous throughout the brain. The subcellular localization of MFSD14A and MFSD14B was further studied using primary mouse embryonic cortex cultures prepared from embryos at day 14–16. Double staining with the Pan neuronal marker, staining axons, dendrites, nucleus and cell body of neurons, confirmed localization of both transporters to neurons (**Figures 6A,D**), as revealed using ELYRA super resolution microscopy (Zeiss). Not all Pan positive cells were positive for the transporters. Neither MFSD14A nor MFSD14B seemed to localize in the plasma membrane as no spatial overlap was seen with the Pan neuronal antibody. As the staining pattern for MFSD14A revealed a more condensed intracellular localization, co-staining with intracellular organelle markers for Golgi was performed. Anti-Giantin antibody, targeting a protein located in the cisterna of the Golgi apparatus (Linstedt and Hauri, 1993; Koreishi et al., 2013), overlapped well with MFSD14A staining (**Figure 6B**). Since MFSD14B had a more spread out staining pattern, co-staining with an anti-KDEL antibody was performed. The anti-KDEL antibody targets the signal peptide for retention and retrieval of protein to the ER (Raykhel et al., 2007), and overlap between MFSD14B and KDEL was seen throughout the cell (**Figure 6E**). MFSD14A also overlapped in some extent to the anti-KDEL staining but only around the nucleus of the cell (**Figure 6C**).

### Gene Expression Changes of Both Transporters after Altered Nutrient Availability

**FIGURE 7:**
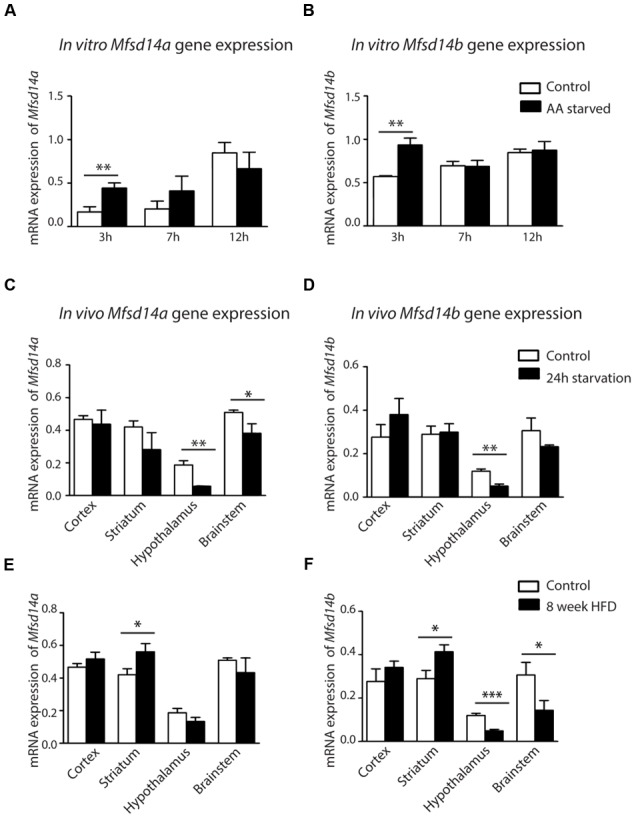
**mRNA expression changes of *Mfsd14A* and *Mfsd14B* in cells and tissue samples.** Mouse cortex primary cultures from e14.5-15 were subjected to amino acid starvation and the mRNA levels monitored at 3, 7 and 12 h. Normalization was performed to three housekeeping genes (*Gapdh, H3a* and *Actb*). The relative mRNA expression (±SD) was plotted, with the highest relative expression set to 100%. Expression differences were calculated using *t*-test and Bonferroni corrected significance levels (^∗^*p* < 0.0493, ^∗∗^*p* < 0.00998, ^∗∗∗^*p* < 0.001). **(A)** *Mfsd14a* expression in amino acid starved cells (AA starved) as compared to control cells and **(B)** *Mfsd14b* expression compared to control cells. Upregulation can be seen for both transporters after 3 h of amino acid starvation. Upregulation of both transporters is also seen in the control cells over time. **(C)** Brain tissue from cortex, striatum, hypothalamus, and brainstem was taken from mice placed on 24 h starvation prior to dissection (*n* = 4, pooled sample). Gene expression was normalized to three housekeeping genes (*Gapdh, H3a* and *Actb*). Relative gene expression levels (±SD) of *Mfsd14a* were compared to normal fed controls, with gene expression of the controls set to 100%. Down regulation was seen in the hypothalamus and brainstem. **(D)** Relative gene expression of *Mfsd14b* after starvation compared to controls. Down regulation was seen in the hypothalamus. **(E)** Brain tissue from mice placed on high fat diet (HFD) for 8 weeks from cortex, striatum, hypothalamus and brainstem was taken (*n* = 4, pooled sample) and used for analysis. Relative gene expression levels (±SD) were compared to normal fed mice. *Mfsd14a* was upregulated in the striatum samples from mice placed on the HFD. **(F)** *Mfsd14b* was upregulated in striatum but downregulated in both hypothalamus and brainstem compared to normal fed controls. Analysis was performed with t-test and Bonferroni corrected significance levels (^∗^*p* < 0.0493, ^∗∗^*p* < 0.00998 and ^∗∗∗^*p* < 0.001).

Amino acid deprivation induced transient upregulation of both *Mfsd14a* and *Mfsd14b* in embryonic primary cortex cultures. Gene regulation was studied in e14-16 primary cortex cultures, cultured in amino acid deprived media for 3, 7, and 12 h. The gene expression of both *Mfsd14a* and *Mfsd14b* was analyzed using qRT-PCR and expression was compared to controls cultured in media complemented with amino acids. After 3 h of amino acid starvation, both *Mfsd14a* and *Mfsd14b* significantly increased relative expression (*P* = 0.0050 and *P* = 0.0014, respectively) compared to controls (**Figures 7A,B**). After 7 h, the gene expression levels of both transporters were at the same levels as the controls and this held true in the 12 h samples as well. As the highest expression value of all samples was set to 100%, an increase in both *Mfsd14a* and *Mfsd14b* can be seen in the control cells over time as well. This would have been overlooked by only comparing control vs. starved at each time point. There is a significant increase of *Mfsd14a* in the 12 h control cells (*P* = 0.0011) compared to the controls at 3 h. The same holds true for the *Mfsd14b* expression, where the relative gene expression increases steadily from 7 h (*P* = 0.0130) through 12 h (*P* = 0.0003) compared to the expression at 3 h in the control cells. Hence, these is an increase in gene expression both due to amino acid starvation at 3 h compared to controls, but also an increase in expression over time in the control cells.

As a change in gene regulation was seen in the primary embryonic cortex cultures, the genes expressions were also analyzed from *in vivo* cortex tissue samples from adult mice subjected to 24 h starvation. To broaden the investigation, samples from striatum, hypothalamus and brainstem were also collected and analyzed. In the cortex samples from adult mice, no significant gene expression changes could be seen for either transporter after a 24 h starvation period. However, a significant downregulation of *Mfsd14a* was found in both hypothalamus (*P* = 0.0029) and brainstem (*P* = 0.0214) (**Figure 7C**). *Mfsd14b* was also significantly downregulated in hypothalamus samples (*P* = 0.0042) (**Figure 7D**). Taken together, nutrient deprivation of primary cortex cultures for 3 h induced a transient induction of *Mfsd14a* and *Mfsd14b* gene expression *in vitro*. However, in *in vivo* cortex samples from mice subjected to 24 h starvation neither transporter was affected but downregulation was found in the hypothalamus and, for *Mfsd14a*, also in the brainstem.

In order to gain insight of how excess nutrition would affect gene regulation *in vivo*, samples from mice subjected to 8 weeks of HFD were taken. At the time of dissection, the mice in the HFD group had gained 38% ± 9% and the control mice on regular chow had gained 12% ± 2.3% as previously described in Perland et al. (2016). Here, a significant upregulation of both *Mfsd14a* and *Mfsd14b* was found in striatum samples (*P* = 0.018 and *P* = 0.012, respectively) (**Figures 7E,F**). In addition, a downregulation in hypothalamus (*P* = 0.0034) and brainstem samples was found for *Mfsd14b* (*P* = 0.046) (**Figure 7F**). Both transporters were hence affected by both starvation and HFD in mice, with *Mfsd14b* more affected by HFD in the samples we dissected out.

## Materials and Methods

### Phylogenetic Analysis and Bioinformatics

The human proteins sequences for MFSD14A and MFSD14B were downloaded together with SLC amino acid sequences of MFS type (SLC2, 15, 16, 17, 18, 19, 22, 29, 33, 37, 40, 43, 45, 46, and SLCO) from ENSEMBL (Cunningham, Release 82), and combined into a multiple PSI/TM sequence alignment using tcoffee (Notredame et al., 2000). Their interrelationships were then analyzed by using the Bayesian approach as implemented in mrBayes 3.2.2 (Huelsenbeck et al., 2001) The analysis was run via the Beagle library (Ayres et al., 2012) on a NVIDA 980Ti graphics card. It was run on six chains (five heated and one cold) with two runs in parallel (*n* runs = 2) under the mixed amino acid model with eight gamma categories and invgamma as gamma rates for a total of 2,000,000 generations. Consensus trees were calculated using the best 75% of the trees generated.

Pairwise protein sequence alignments were calculated using EMBOSS Needle for global sequence similarities and EMBOSS Water, using the Smith–Waterman algorithm, for local similarities between proteins^3^ (Rice et al., 2000). FASTA sequences were retrieved from NCBI. For MFSD14A, sequences from accession number NP_149044.2 (human) and NP_032272.2 (mouse) were used. For MFSD14B, sequences from accession number NP_115947.2 (human) and NP_001077370.1 (mouse) were used.

Glycosylation predictions were performed using the NetOGlyc 4.0 Server from CBS Predictions Servers (Steentoft et al., 2013). Here, the mouse FASTA protein sequences used above for each protein were entered.

### Animals

All procedures involving mice were approved by the local ethical committee in Uppsala (Uppsala Djurförsöksetiska Nämnd, Uppsala District Court, permit number C39/16, C67/13 and C419/12) in unity with the guidelines of European Communities Council Directive (2010/63). All procedures involving mice were planed and performed in such a way as to minimize suffering. Euthanasia was performed during the light period by either cervical dislocation or transcardiac perfusion. All animals were maintained in a temperature controlled room on a 12 h light-dark cycle where they had free access to water and food unless otherwise stated. C57Bl6/J (Taconic M&B, Denmark) females and males were used and considered as wildtype.

### Sample Collection from Mice for RNA Extractions and cDNA Synthesis

Wild type mice panels and mouse diet experiment were performed and prepared as described in Perland et al. (2016). All tissues were kept in RNA-later (Qiagen, USA) for 2 h at room temperature before placed at -80°C until RNA was extracted. Blood was collected via cardiac puncture and mixed with EDTA (VWR) at 1.5 mg/ml blood before centrifuged (Heraesus Fresco 21 centrifuge, Thermo Scientific) for 10 min at 4°C, 14.8x 1000 min^-1^; the pellet was used for RNA extraction. The RNA was retrieved from individual samples using Absolutely RNA Miniprep Kit (Agilent Technologies), according to the manufacturer’s instructions. Concentrations were measured using ND-1000 spectrophotometer (NanoDrop Technologies). Two micrograms RNA template was used for the cDNA synthesis, performed according to manufacturer recommendations using the Applied Biosystems High Capacity RNA-to-cDNA kit (Invitrogen). cDNA from the same region, but from different animals, were pooled and diluted to a concentration of 5 ng/μl.

### Primer Design and Quantitative Real-Time PCR (qRT-PCR)

Gene expression and expression changes were determined using qRT-PCR. All primers were designed using Beacon Design 8 (Premier Biosoft). *Mfsd14a* forward 5′-gcttcttcttatacctcagaca-3′, reverse 5′-atgccaaggactgctatg-3′, *Mfsd14b* forward 5′-ctgaaaactactcactgcca-3′, reverse 5′-gtgcaggaagcgaacagg-3′. Reference housekeeping genes used were : glyceraldehyde-3-phosphate dehydrogenase (*Gapdh*) forward 5′-gccttccgtgttcctacc-3′, reverse 5′-gcctgcttcaccaccttc-3′, beta tubulin 4B (*bTub*)forward 5′-agtgctcctcttctacag-3′, reverse 5′-tatctccgtggtaagtgc-3′, ribosomal protein L19 (*Rpl19*) forward 5′-aatcgccaatgccaactc-3′, reverse 5′-ggaatggacagtcacagg-3′, histone cluster 1 (*H3a)* forward5′-ccttgtgggtctgtttga-3′, reverse 5′-cagttggatgtccttggg-3′, peptidylpropyl isomeras A (*Cyclo*) forward 5′-tttgggaaggtgaaagaagg-3′, reverse 5′-acagaaggaatggtttgatgg-3′ and actin-related protein 1B (*Actb*) forward 5′-ccttcttgggtatggaatcctgtg-3′, reverse 5′-cagcactgtgttggcatagagg-3′. Final volume for each qRT-PCR reaction was 20 μl consisting of: 1 μl pooled cDNA (5 ng/μl), 0.05 μl of each primer (100 pmol/μl), 3.6 μl 10x DreamTaq buffer (Thermo Fisher Scientific), 0.2 μl of 25 mM dNTP mix (Thermo Fisher Scientific), 1 μl DMSO, 0.5 μl SYBR Green (Invitrogen) and 0.08 μl of Dream Taq (5 U/μl, Thermo Fisher scientific). The volume was adjusted to 20 μl with sterile water. An iCycler real-time detection instrument (Bio-Rad) was used with the following settings: initial denaturation for 30 s at 95°C, 50 cycles of 10 s at 95°C, 30 s at 55–61°C (optimal temperature depending on primer) and 30 s at 72°C. A melting curve was performed starting at 55°C for 81 cycles at 10 s interval and a temperature increase of 0.5°C per cycle. All qRT-PCR were run in triplicates and a negative control was included on each plate. The qRT-PCR was run twice. All data was collected using the MyIQ (Bio-Rad Laboratories) software.

### Analysis of qRT-PCR Data

Primer efficiency was calculated using the LinRegPCR software, followed by Grubbs test (GraphPad software) to remove outliers before calculations were corrected for each primer based on primer efficiency. The GeNorm protocol (Vandesompele et al., 2002) was used to check for stable housekeeping genes (*M* < 1.5) and the expression was then normalize using Geomean toward the most stable housekeeping genes. The qRT-PCRs were run twice for each target and representative results from one run presented. The corrected and normalized *C*_t_ values were plotted (±SD) for each gene. Statistical analyses were performed in Graph Pad Prism version 5. *T*-tests were performed for gene expression changes with Bonferroni corrected significance level where ^∗^*p* < 0.0493, ^∗∗^*p* < 0.00998, and ^∗∗∗^*p* < 0.001.

### Western Blot

Protein samples from mouse brain tissue was prepared as described in Perland et al. (2016). SDS-PAGE was performed using 20 μg of protein sample diluted 1:1 in Laemmlis buffer (Bio-Rad Laboratories) with β-Mercaptoethanol (Sigma-Aldrich) and heat treated at 97°C for 8 min. Gel separation with a 12% TGX Miniproteina gels (Bio-Rad Laboratories), run at 150 V for 40 min, was performed before blotting onto PVDF membrane (Immobilon-P, Millipore) at 50 V for 60 min on ice. A molecular weight marker was used as a reference (PageRule Prestained, Thermo Fisher Scientific). The membrane was blocked in 5% milk TTBS blocking solution for 60 min. The membrane and primary antibodies HIAT1 (MFSD14A) (ab103836, Abcam) diluted 1:200 and HIATL1 (MFSD14B) (SAB2107506, Sigma-Aldrich) diluted 1:100 were incubated overnight at 4°C. After 3x 10 min wash with TTBS, the membrane was incubated at room temperature for 60 min with HRP coupled secondary antibodies, HRP goat α-rabbit, diluted 1:10000 (Invitrogen). The membrane was developed using Clarity Western ECL Substrate (Bio-Rad) and visualized using a CCD camera (ChemiDoc, Bio-Rad) and staining was compared to the molecular weight marker using Image Lab Software 5.2.1 build 11 (Bio-Rad).

### Non-fluorescent Free Floating DAB-Immunohistochemistry

Brain sections, 70 μm, from two male mice were used. All chemicals were purchased from Sigma-Aldrich, unless otherwise stated. The sections were washed in TBS 4x 8 min, and for brains stained with anti-HIAT1 antibodies, a citric acid antigen retrieval step was performed for 40 min in 0.01 M citric acid (pH 6.0) at 70°C before proceeding. Then all brain sections were rinsed in TBS 4x 8 min before and after incubation in 10% MeOH, 3% H_2_O_2_ (Merck Millipore) in TBS for 10 min. Sections were then incubated in 1X blocking reagent (Roche Diagnostics) for 1 h followed by incubation in HIAT1, diluted 1:1000, or HIATL1, diluted 1:500, diluted in supermix (0.25% gelatin, 0.5% Triton X-100 in TBS) overnight at 4°C. Sections were rinsed in TBS 2x1 + 4x8 min followed by incubation in the secondary antibody (biotinylated goat-anti-rabbit IgG (H+L), Vector laboratories, USA) diluted 1:400 in supermix for 1 h. Sections were rinsed in TBS 5x 8 min before and after incubation in ABC kit (Reagent A, Reagent B (Vectastain, Vector Laboratories), and diluted 1:800 in supermix for 1 h. Sections were incubated in 0.05% DAB (3,3′-diaminobenzidine) tetrahydrochloride, 0.35% NiCl and 0.015% H_2_O_2_ and rinsed 4x 5 min in TBS and mounted on gelatinized microscope slides (Menzel Gläser, Germany). Sections were dehydrated for 5 min in 70% and 95% EtOH, 10 min in 100% EtOH (Solveco) and 20 min in Xylene. Slides were mounted in DPX Mountant for histology with micro cover slides Superfrost Plus (Menzel Gläser, Germany). Sections were analyzed using a Mirax Pannoramic midi scanner with the Pannoramic Viewer software version 1.15.4 RTM (3dHistech).

### Fluorescent Immunohistochemistry on Mouse Brain Tissue

Brain tissue for immunohistochemistry was collected as described in Roshanbin et al. (2014). Fluorescent immunohistochemistry on paraffin embedded mouse brain sections was performed as described in Perland et al. (2016) with antibodies all diluted in supermix for HIAT1, diluted 1:100, or HIATL1, diluted 1:50, NeuN (Merck Millipore) diluted 1:200, GFAP (Abcam) diluted 1:800 and nucleus marker DAPI (Sigma Aldrich) diluted 1:5000 in PBS. Images were acquired using a fluorescent microscope Olympus microscope BX53 with an Olympus DP73 camera. The micrographs were acquired by CellSens Dimension software.

### Fluorescent Immunocytochemistry on Mouse Primary Cortex Cultures

Wildtype male and female mice were mated and at e14-15 the females were sacrificed, the embryos removed and cortex dissected out and primary cultures were set up as previously described in Perland et al. (2016).

Immunocytochemistry was performed as described in Perland et al. (2016) with anti-HIAT1 diluted 1:100, or anti-HIATL1 diluted 1:50 in supermix blocking solution. Co-staining with neuronal marker Pan diluted 1:200 (MAB2300, Millipore), Golgi markers Giantin (ab37266, Abcam), and KDEL markers (ab12223, Abcam) all diluted 1:200 in supermix blocking solution. Images were acquired at the SciLifeLab BioVis Facility (Uppsala University) using super resolution microscopy (Zeiss ELYRA S.1) and the Zen black software (Zeiss) and images were then handled using ImageJ, Fiji edition (Schindelin et al., 2012).

### Amino Acid Starvation Experiments in Primary Cortex Cells

To evaluate changes in gene expression of *Mfsd14a* and *Mfsd14b* in response to nutrient availability, primary embryonic cortex cultures were deprived of common amino acids. At culture day 10, cells were subjected to amino acid starvation. EBSS media (Gibco) media was prepared to mimic the Neurobasal A media, that the cultures were grown in, by adding 1.0 mM Sodium-Pyruvate, 1% Penicillin-Streptomycin, 2% B-27 (50X), 4X MEM Vitamin Solution (100X) (Gibco) and 10.9 mM HEPES (1 M) buffer solution (Gibco) and amino acids L-arginine, L-cysteine, L-lysine, L-methionine, L-phenylalanine, L-proline, L-threonine, L-tryptophan, and L-tyrosine (Sigma-Aldrich). To the control media 2.0 mM GlutaMax and additional amino acids were added: glycine, L-alanine, L-asparagine, L-histidine, L-isoleucine, L-leucine, L-serine, and L-valine (Sigma-Aldrich). The starved cells were hence deprived of the following amino acids, glycine, L-alanine, L-asparagine, L-glutamine, L-histidine, L-isoleucine, L-leucine, L-serine, and L-valine. The experiment was run in triplicates in each treatment group (starved versus control cells) and the cells were treated in the limited amino acid medium or the complete amino acid medium for 3, 7 or 12 h before RNA was extracted with RNeasy Midi Kit (Qiagen, Germany), following the manufactures protocol. cDNA synthesis and qPCR using 2 μl cDNA (30 ng/μl) were run and analyzed as described above. The stable housekeeping genes *Gapdh, H3a*, and *Actb* were used as reference genes. Corrected and normalized gene expression values were plotted as relative expression (±SD) with the highest expression set to 100% in GraphPad Prism 5 Software and unpaired *t*-test was conducted to check for significant changes.

### Diet Experiments in Adult Mice

Diet experiments were set up as described in Perland et al. (2016) and material was used here to analyze the expression patterns of *Mfsd14a* and *Mfsd14b*. Briefly, adult male mice were starved for 24 h before euthanasia. Brain regions (cortex, striatum, hypothalamus, and brainstem) from four mice per group were dissected out, RNA extractions and cDNA synthesis was performed as detailed above and the samples pooled before qRT-PCR. Mice were also placed on a high fat western diet (HFD) consisting of 21% fat by weight, 17.2% protein, 43% carbohydrates (fed R638, Lantmännen) while control mice were kept on standard chow consisting of 5% fat, 21% protein, and 51.5% carbohydrates (R3, Lantmännen), for 8 weeks. Induced obesity was monitored by weighing the mice in the control group as well as the HFD mice every 2 weeks. The HFD mice were significantly heavier than the controls at the day of dissection (Perland et al., 2016). Four mice per group were used for brain region analysis of gene regulation (cortex, striatum, hypothalamus, and brainstem).

## Discussion

Here, we have characterized two putative transporters, MFSD14A and MFSD14B (also known as HIAT1 and HIATL1, respectively), which phylogenetically cluster to the SLCs of MFS type. However, they differed phylogenetically to such an extent that, when fully classified, they will most likely be grouped into a new SLC family, and not into any of the 52 existing SLC families. The two putative transporters were found to be similar in sequence to each other and high sequence similarity was also found between human and mouse protein sequences. The phylogenetical analysis was performed on human SLCs of MFS type as the end goal is to better understand the relationships and functions of human transporters. The high sequence similarity found between mouse and human proteins opens up the possibility of shared function in both species. The gene expression analysis performed here in mice, corroborate previous gene expression panels performed in rats (Sreedharan et al., 2011), and expression could be found in both peripheral tissues and in the central nervous system indicating that the genes are, or have been, vital. Recently, *Mfsd14a* was found to be involved in spermatogenesis, as the lack of the transporter resulted in failed acrosome formation, sperm head condensation, and faulty mitochondrial localization (Doran et al., 2016). *Mfsd14a* expression was located to the supportive Sertoli cells, where the authors suggested they be responsible for sugar uptake (e.g., mannose) important for glycosylation of key molecules required for acrosome formation. While the knock down of *Mfsd14a* did not directly cause death to the animals, the males were infertile, making this transporter vital for reproduction and hence the survival of the species. In addition, since only *Mfsd14a* was knocked down in this strain, and not *Mfsd14b*, the functions of these two proteins are probably not the same as a clear phenotype was seen. We can here show that there are clear differences in protein localization throughout the mouse brain, intracellular localization and that the transporters have different responses to starvation and HFD, and it is therefore plausible that MFSD14A and MFSD14B transport different substrates and/or conduct their transport in different organelles and cells.

On a protein level, MFSD14A staining was found ubiquitously throughout the mouse brain, and the staining patterns of MFSD14A correlates well with expression patterns of other neuronal transporters (Dahlin et al., 2009) found when studying the expression of 307 *Slc* genes in the Allen Brain Atlas^4^ and comparing the patterns to neuronal, interneuronal, and astrocytic markers. The ubiquitous protein expression found here also correlates with transcript expression data from neonatal mouse brains (Matsuo et al., 1997) where high expression was found from embryonic day 13 through adulthood. MFSD14B staining was found to be more spatially spread out than MFSD14A and correlates with some of the *slc* genes expressed in interneurons in the study by Dahlin et al. As neither MFSD14A nor MFSD14B was among the 307 *Slc* genes included in the screen, our panels adds information to the expression patterns of transporters in the mouse brain. Co-localization to neurons was verified as both MFSD14A and MFSD14B staining localized to cells stained with the neuronal, NeuN, antibody which recognizes a neuron specific nuclear protein in vertebrates (Mullen et al., 1992), but not with cells positive for the astrocyte marker GFAP (**Figure 5**). Their intracellular placement was studied with markers for Golgi and the ER and the intracellular localization placed MFSD14A in and around the ER and Golgi complex and MFSD14B possibly in the ER. The Golgi complex is a central organelle in the endomembrane system, with a well-studied role in processing and sorting of transmembrane and soluble proteins received from the ER on their way to their final destination. Recently, new functions have been attributed to the Golgi, making this organelle an active component in pathways controlling mitotic energy, cytoskeleton organization, calcium homeostasis and even plasma membrane receptor-initiated signaling events that respond to growth signals and energy status (Wilson et al., 2011). In addition, the intracellular placement of MFSD14A to the Golgi is hypothesized in the work done by Doran et al (Doran et al., 2016) due to the failed acrosome formation in their infertile knock out mice. The acrosome is a highly conserved membranous organelle located over the sperm nucleus, and active trafficking from the Golgi apparatus is known to be involved in acrosome formations, although many molecular mechanisms controlling this event are still unknown (Berruti and Paiardi, 2011). MFSD14A has been showed to contain a conserved sugar transporter motif (Matsuo et al., 1997) and while the function of this transporter is still unknown, involvement in glycosylation events in the Golgi or ER seems plausible and the disrupted glycosylation could be the culprit for the faulty spermatogenesis found in the knock out mice used in the study above.

That *Mfsd14a* and *Mfsd14b* gene expression was found to be temporarily upregulated in primary cortex cells subjected to amino acid starvation (**Figures 7A,B**) was initially surprising, as internal membrane proteins are spatially separated from directly sensing extracellular nutrient availability. As MFSD14A and MFSD14B could be involved in the functions of Golgi and ER, due to their intracellular localization, changes in gene expression could possibly be due to alterations in the glycosylation processes. The Golgi machinery has been found to be an important part in integrating nutrient-sensing signals with cell fate (Lau et al., 2007). One other interesting finding is that the expression of both transporters increased in the control cells over time. As the housekeeping genes were stable, the gene regulation effects seen could be due to general expressional changes in cells as the nutrient in the media is used up or as the cells acclimatize after a media change. In this study, we also found that the transporters were affected in mouse brain regions after starvation and HFD. Effects in the hypothalamus, a coordination center for neurons affected by peripheral hormones and signals connected to feeding and energy metabolism (van den Top et al., 2004; Betley et al., 2015) was seen for both transporters after starvation and could further highlight their possible role in cellular metabolism. In addition, a HFD also induced changes in the hypothalamus for *Mfsd14b*, but not for *Mfsd14a*. The HFD did cause upregulation of both transporters in striatum, an area involved in motivation and reward (Yager et al., 2015). *Mfsd14b* was additionally downregulated in hypothalamus and brainstem. Due to the high energy demand of the brain and its central role in neurotransmission, an arsenal of transporters are utilized to supply nutrients, facilitate energy production, control ion and pH balance, regulate both neurotransmitter levels and activity, and not the least, remove metabolic by-products (Simpson et al., 2007; Dahlin et al., 2009). While the nutritional availability was the intended experimental model, other obesity linked factors, such as hyperinsulinemia, hyperglycemia, and hypertension have previously been shown to occur in the mouse strain, C57BL/6J, used in these experiments (Collins et al., 2004), hence, the gene regulation changes seen could possibly be due to other confounding factors and not just energy metabolism.

Even though the functions of MFSD14A and MFSD14B still are unknown, the intracellular localization of the transporters in neurons and their change due to energy availability makes these transporters highly interesting. The protein expression of these transporters in the mouse brain and cellular localization has, to our knowledge not been studied before. The gene regulation results seen for both transporters, both *in vitro* and *in vivo*, depending on nutrient availability warrants more research as both clearly are involved in regulation of important cellular pathways and the involvement of MFSD14A in spermatogenesis (Doran et al., 2016), provides a clear example why these SLCs are important to study further.

## Ethics Statement

This study was carried out in accordance with the recommendations of and the guidelines of the European Communities Council Directive (2010/63). All procedures involving mice were approved by the local ethical committee in Uppsala, Uppsala Djurförsöksetiska Nämnd, Uppsala District Court, permit number C39/16, C67/13 and C419/12.

## Author Contributions

EL designed experiments, prepared material and performed immunohistochemistry, immunocytochemistry, qRT-PCR, and analysis, set up diet experiments and primary cultures, imaging, complied figures and tables and drafted the manuscript. EP set up diet experiments and primary cultures, aided in analysis of results, finalized figures for bioinformatics and DAB immunohistochemistry. ME performed RNA extractions, cDNA synthesis, designed and optimized primers and qRT-PCR and analyzed data. SH planned and performed starvations experiments in primary cultures, prepared tissue for and performed non-fluorescent DAB immunohistochemistry, as well as aided in the analysis of these results. FL performed RNA extractions, cDNA synthesis and immunostainings. JR performed antibody optimization and initial immunohistochemistry. RF designed the project, performed bioinformatics and analysis. All authors have read and approved of the whole manuscript and helped with interpretation of results.

## Conflict of Interest Statement

The authors declare that the research was conducted in the absence of any commercial or financial relationships that could be construed as a potential conflict of interest.

## Footnotes

1 http://slc.bioparadigms.org/

2 http://emboss.open-bio.org/

3 http://emboss.open-bio.org/

4 http://www.brain-map.org
